# Association of cardiovascular disease prevalence with BMD and fracture in men with T2DM

**DOI:** 10.1111/1753-0407.13530

**Published:** 2024-04-07

**Authors:** Xiao‐ke Kong, Rui Xie, Deng Zhang, Xiao‐jing Chen, Xiao‐feng Wang, Jie‐li Lu, Hong‐yan Zhao, Jian‐min Liu, Li‐hao Sun, Bei Tao

**Affiliations:** ^1^ Department of Endocrine and Metabolic Diseases, Shanghai Institute of Endocrine and Metabolic Diseases Ruijin Hospital, Shanghai Jiao Tong University School of Medicine Shanghai China; ^2^ Shanghai National Clinical Research Center for Metabolic Diseases, Key Laboratory for Endocrine and Metabolic Diseases of the National Health Commission of the PR China, Shanghai Key Laboratory for Endocrine Tumor, State Key Laboratory of Medical Genomics Ruijin Hospital, Shanghai Jiao Tong University School of Medicine Shanghai China

**Keywords:** bone mineral density, cardiovascular disease, type 2 diabetes mellitus

## Abstract

**Background:**

Patients with type 2 diabetes mellitus (T2DM) are predisposed to cardiovascular disease (CVD). Bone mineral density (BMD) is linked to CVD, but most studies focused on women. Our analysis aims to explore the association of BMD and fracture with the prevalence of CVD in men with T2DM.

**Methods:**

In this retrospective cross‐sectional study, 856 men with T2DM were enrolled. BMDs at the lumbar spine (L2‐4), femoral neck (FN), and total hip (TH) were measured by dual‐energy X‐ray absorptiometry (DXA). The CVD outcome was determined as the sum of the following conditions: congestive heart failure, coronary heart disease, angina pectoris, myocardial infarction, the requirement for coronary artery revascularization, and stroke. The relationship between BMDs and CVD was investigated by restricted cubic spline curves and logistic regression models.

**Results:**

A total of 163 (19.0%) patients developed CVD. The restricted cubic spline curve revealed a linear and negative association between FN‐BMD, TH‐BMD, and CVD. After full adjustments for confounding covariates, the odds ratios were 1.34 (95% confidence interval [CI] [1.11–1.61], *p* < .05), 1.3 (95% CI [1.05–1.60], *p* < .05), and 1.26 (95% CI [1.02–1.55], *p* < .05) for each 1‐SD decrease in BMDs of L2‐4, FN and TH, respectively. T‐scores of < −1 for BMD of L2‐4 and FN were independently associated with CVD (*p* < .05). Subgroup analyses further supported our findings.

**Conclusions:**

The prevalence of CVD was inversely correlated with BMD levels in men with T2DM, particularly at the FN. We hypothesized that monitoring FN‐BMD and early intervention would help reduce CVD risk in men with T2DM, especially those with hypertension.

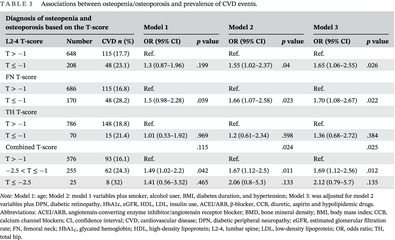

## Graphical abstract

Highlights

- This is the first study to explore the association between bone mineral density, fracture, and the prevalence of cardiovascular disease in men with type 2 diabetes mellitus.
- We have drawn the restricted cubic spline curve and revealed that femoral neck bone mineral density, total hip bone mineral density, and cardiovascular disease have a linear negative correlation.
- The previous fracture was independently associated with an increased risk of cardiovascular disease.

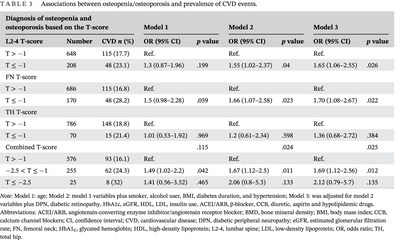

## 1 INTRODUCTION

Type 2 diabetes mellitus (T2DM) is a rapidly growing public health problem. According to the epidemiological survey, the prevalence of T2DM in Chinese adults is 11.2%.^1^ Osteoporosis is a systemic metabolic disease characterized by reduced bone mineral densities (BMDs) and deterioration of bone microstructure, with a consequent increase in bone fragility and susceptibility to fracture.^2^ Cardiovascular diseases (CVDs) are mainly circulatory diseases caused by atherosclerosis, including coronary heart disease, congestive heart failure, and cerebrovascular diseases. More importantly, osteoporosis and CVD are prevalent health problems that seriously affect the quality of life and survival of the elderly with T2DM. Both diseases are characterized by high rates of morbidity, disability, and mortality.^3^, ^4^

Previously, it was believed that the occurrence of osteoporosis and CVD was independent. However, several studies in recent years have shown that low BMDs and fractures are associated with CVD, and people with low BMDs are at an increased risk of developing CVD.^5^ Studies have shown a pathophysiological relationship between osteoporosis and vascular calcification, which involves the pathogenesis of bone morphogenetic protein, RANKL‐RANK‐OPG pathway, wnt signaling pathway, matrix gla protein, and vitamin K.^6^, ^7^ Low BMD is not only a risk factor for osteoporosis, fracture, and death in the general population,^8^ but it also predicts coronary artery disease progression better than traditional risk factors such as hyperlipidemia and smoking.^9^ Given that CVD is the leading cause of premature death in patients with T2DM,^10^ it is critical to understand whether low BMD is an underlying risk factor for CVD in patients with T2DM.

Most previous studies focused more on bone health in women, even though it was estimated that about 39% of new osteoporotic fractures worldwide in 2000 occurred in men.^11^ In particular, the rate of osteoporosis in men was underestimated, as many subjects who experienced fragility fractures did not report BMD impairment.^12^ A study showed a comparable prevalence of osteoporosis between men aged 70 and women over 65 years.^13^ Retrospective investigations have provided compelling evidence of an increased incidence of osteoporosis and hip fracture in older men with diabetes, indicating that diabetes adversely affects bone health in men.^14^ In addition, it has been found that osteoporosis and fractures in men were associated with poor clinical outcomes. In absolute numbers, the prevalence of vertebral or hip fracture in older men was approximately one‐third of that in women, but the mortality rate associated with hip fractures, as well as vertebral and other major fractures, was higher in men than in women.^15^, ^16^, ^17^, ^18^ A large prospective cohort study in Spain found that men with T2DM had an elevated risk of hip fracture compared to those without the condition.

Furthermore, men with T2DM were found to have a 28% higher risk of mortality following a hip fracture.^19^ Several extensive case–control studies conducted in patients with hip fractures have shown that the risk of postoperative cardiac events in diabetic patients was significantly increased compared with nondiabetic patients, and the length of hospital stay was increased by 1–4 days.^20^ This suggests that the relationship between CVD and fractures in men with T2DM is worth exploring.

Therefore, the purpose of this study is to explore the relationship between BMDs, osteoporotic fracture, and the prevalence of CVD in Chinese men with T2DM. We hypothesize that low BMD could be used as a reference indicator to alert subjects at high risk of CVD in men with T2DM.

## 2 METHODS

### 2.1 Participants

This was a cross‐sectional, retrospective, single‐center study. This study extracted data from the electronic health records (EHRs) of men with T2DM from the Department of Endocrine and Metabolic Diseases, Rui‐jin Hospital, Shanghai Jiao Tong University School of Medicine, Shanghai, China, between August 2009 and July 2013. Each patient had a unique identification number, and the first record was selected if multiple medical records were available for the same individuals. Participants with diseases that may severely influence bone mass and metabolism were excluded, such as end‐stage renal disease (estimated glomerular filtration rate [eGFR] <30 mL/min/1.73 m^2^), hyperparathyroidism, hypoparathyroidism, malignant tumors, and Cushing's syndrome. Then, after removing incomplete BMD data or participants younger than 40 years of age, a total of 856 men with T2DM were available for our study.

The Ethics Committee of Rui‐jin Hospital, Shanghai Jiao‐tong University School of Medicine approved our study. This research was retrospectively registered in the Chinese Clinical Trials Registry (ChiCTR2100050913).

### 2.2 Clinical characteristics

The standardized self‐administered questionnaires about sociodemographic characteristics, medication records, and any medical or surgical histories were conducted by trained residents. The body mass index (BMI) was calculated as the body weight divided by the squared height (kg/m^2^). Diabetes duration was self‐reported by the patients, and insulin use was identified in the EHRs based on the patients' hypoglycemic agents at discharge. Diabetic peripheral neuropathy (DPN) was comprehensively examined and diagnosed based on the patient's neurological function examination and clinical symptoms. Diabetic retinopathy was diagnosed based on fundus examination (any grade detected by ophthalmoscopy and ophthalmologist assessment). Hypertension was self‐reported or diagnosed in the medical records. Medication use (including lipid‐lowering medication and antihypertensive medication) was obtained from the EHRs. Patients fasted for at least 10 h before morning blood collection. Glycated hemoglobin A1c (HbA1c) was measured using a hemoglobin testing system (Variant II, Bio–Rad, Hercules, CA). Total cholesterol, triglycerides, low‐density lipoprotein‐cholesterol, high‐density lipoprotein‐cholesterol, and creatinine levels were measured using an automatic biochemical analyzer (Modular E170, Roche, Basel, Switzerland). The eGFR was calculated using the Chronic Kidney Disease Epidemiology Collaboration equation.

### 2.3 BMD measurement

BMDs at the lumbar spine (L2‐4), femoral neck (FN), and total hip (TH) were measured by dual‐energy X‐ray absorptiometry (DXA, Lunar Expert‐1313, Lunar Corp, Madison, WI). The T‐score was calculated as an individual's BMD, which was compared with the mean value of a young, healthy reference population, and the difference was represented as an SD.

### 2.4 Previous fracture and CVD ascertainment

Questionnaires recorded basic information about the previous fracture in the EHRs. Participants were asked: “Has a doctor ever told you that you had broken or fractured bones?” Fractures of the fingers, toes, and skull/face were excluded. Besides, fractures associated with trauma or that occurred in childhood were not considered osteoporotic fractures. The CVD outcome was defined as the sum of the following six outcomes: congestive heart failure, coronary heart disease, angina pectoris, myocardial infarction, the need for coronary artery revascularization, and stroke, and they were identified by self‐reported and diagnosis records in the EHRs.

### 2.5 Statistical analysis

All analyses were conducted using the IBM SPSS Statistics 25 (version 9.2) and R (version 4.0.5). Continuous variables were presented as the mean ± SD for normal distributions and median (interquartile range) for skewed distributions. Independent‐sample *t*‐tests and Mann–Whitney *U* tests were used to compare the two groups. Categorical variables were expressed as percentages, and chi‐square (*χ* ^2^) tests were used to compare these values between the two groups.

**FIGURE 1:**
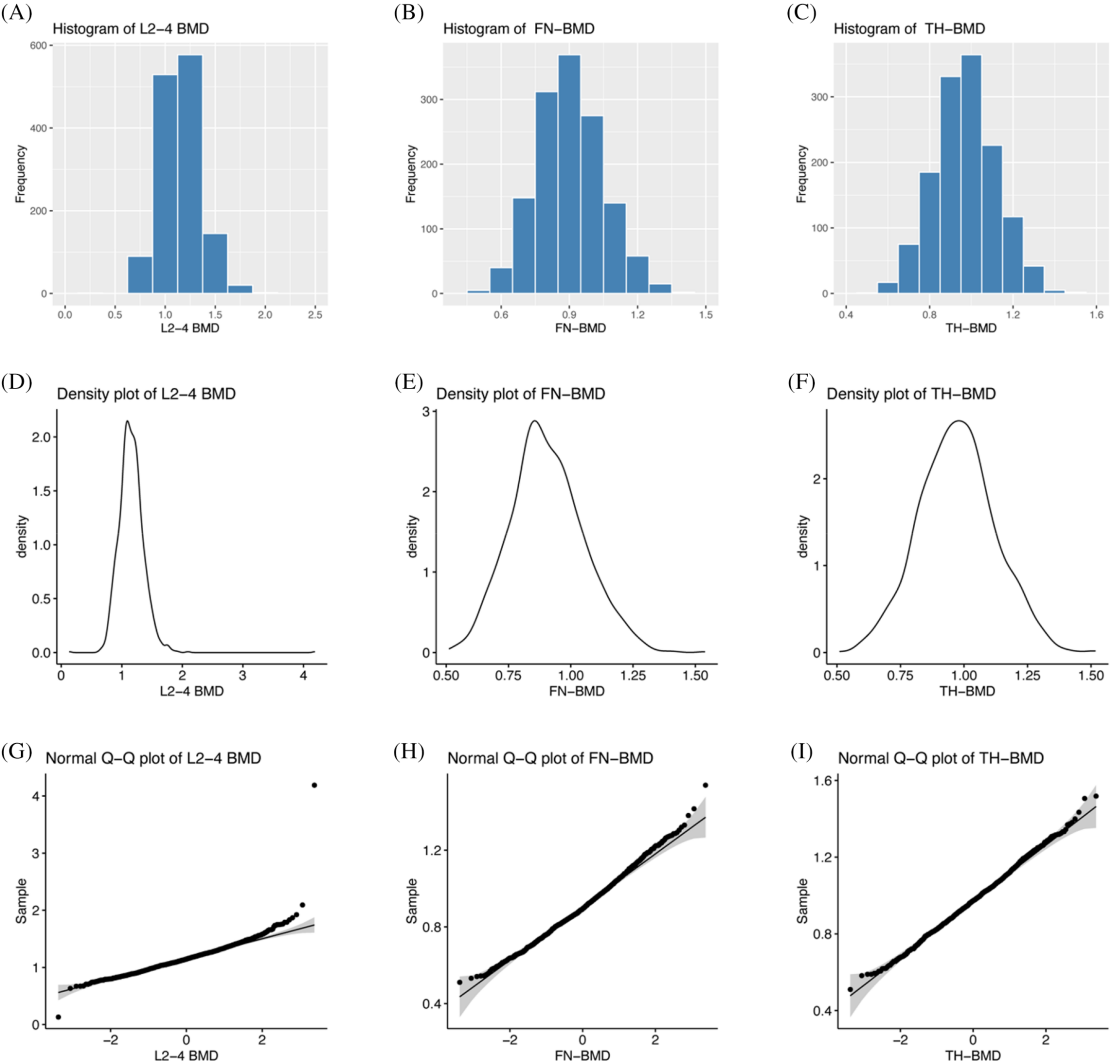
(A–I) BMD normal distribution test diagram, including histogram, density plot, and Q‐Q plot. (A–C) The histogram of L2‐4BMD, FN‐BMD, and TH‐BMD. (D–F) The density plot of L2‐4BMD, FN‐BMD, and TH‐BMD. (G–I) The Q‐Q plot of L2‐4BMD, FN‐BMD, and TH‐BMD. BMD, bone mineral density; CVD, cardiovascular disease; FBMD, femoral neck bone mineral density; FN, femoral neck; LBND, lumbar spine bone mineral density; TBMD, total hip bone mineral density; TH, total hip.

BMD distribution is approximately normal (Figure 1A–I). In order to avoid the problem of multicollinearity, the correlation between variables was checked by observing the value of the coefficient of the variance inflation factor. In multiple logistic regression analysis, we started with 21 variables and excluded 2 variables with variance inflation factor >5. The restricted cubic spline curve was used to explore the linear relationship between different measurement sites of BMD and CVD events. The logistic regression model was used to estimate odds ratios (ORs) for CVD events, according to the per 1‐SD decrease in BMD for each measurement site (lumbar spine, FN, and TH). A clinical diagnosis of osteopenia/osteoporosis was based on the BMD T‐score for each measurement site or the combined T‐score of the three measurement sites. First, model 1 was adjusted for age. Second, model 2 was adjusted for age plus smoking, alcohol drinking, BMI, diabetes duration, and hypertension. Model 3, as our core model, was adjusted for model 2 variables plus DPN, diabetic retinopathy, HbA1c, eGFR, high‐density lipoprotein‐cholesterol, low‐density lipoprotein‐cholesterol, insulin use, angiotensin‐converting enzyme inhibitor/angiotensin receptor blocker, β‐blocker, calcium channel blockers, diuretic, aspirin, and hypolipidemic drugs. Subgroup analyses, stratified by age, BMI, diabetes duration, hypertension, DPN, and diabetic retinopathy, were conducted to evaluate the interactions. Two‐sided *p* values <.05 were considered statistically significant.

## 3 RESULTS

### 3.1 Baseline characteristics of the participants

The baseline characteristics of the study population are shown in Table 1. There were 856 participants in total, 163 of whom were diagnosed with CVD. Age and diabetes duration significantly differed between CVD and non‐CVD individuals, whereas BMI and HbA1c did not differ significantly. The percentages of DPN, diabetic retinopathy, previous fracture, hypertension, and antihypertensive drugs used were significantly higher in the CVD group than in the non‐CVD group, whereas the BMDs (g/cm^2^, T‐score) at FN or TH, and eGFR of the CVD group were significantly lower than those of the non‐CVD group.

**TABLE 1:** Baseline characteristics of the participants stratified by CVD in men.

	Non‐CVD	CVD	*p*
*N*	693	163	
Age (years)	54 (48, 60)	62 (55, 69)	<.001
BMI (kg/m^2^)	25.12 ± 3.33	25.41 ± 3.73	.348
Smoker (%)	308 (44.4)	69 (42.3)	.625
Alcohol user (%)	175 (25.3)	39 (23.9)	.725
Previous fracture (%)	18 (2.6)	14 (8.6)	<.001
Diabetes duration (years)	7 (3, 12)	10 (5, 17)	<.001
HbA1c (%)	8.2 (7, 9.7)	8 (7, 9.38)	.316
DPN (%)	228 (32.9)	82 (50.3)	<.001
Diabetic retinopathy (%)	120 (17.3)	52 (31.9)	<.001
eGFR (mL/min/1.73 m^2^)	98.25 ± 15.01	87.65 ± 19.31	<.001
Total cholesterol (mmol/L)	4.58 ± 1.03	4.22 ± 1.05	<.001
Triglycerides (mmol/L)	2.32 ± 2.34	1.9 ± 1.54	.03
HDL (mmol/L)	1.07 ± 0.3	1.1 ± 0.29	.187
LDL (mmol/L)	2.76 ± 0.83	2.47 ± 0.8	<.001
Hypertension (%)	323 (46.6)	113 (69.3)	<.001
Insulin use (%)	250 (36.1)	69 (42.3)	.137
ACEI/ARB (%)	259 (37.4)	90 (55.2)	<.001
β‐blocker (%)	20 (2.9)	13 (8)	.002
CCB (%)	81 (11.7)	40 (24.5)	<.001
Diuretic (%)	17 (2.5)	9 (5.5)	.04
Aspirin (%)	198 (28.6)	61 (37.4)	.027
Hypolipidemic drugs (%)	317 (45.7)	80 (49.1)	.442
BMD (g/cm^2^)			
L2‐4	1.210 ± 0.185	1.185 ± 0.206	.132
FN	0.949 ± 0.136	0.901 ± 0.131	<.001
TH	1.014 ± 0.139	0.984 ± 0.131	.013
BMD (T‐score)			
L2‐4	0.2 ± 1.49	−0.03 ± 1.71	.093
FN	−0.00 ± 1.04	−0.37 ± 0.98	<.001
TH	0.55 ± 1.07	0.31 ± 1.01	.008

*Note*: The data are summarized as the mean ± SD or median (interquartile range) for continuous variables or as a numerical proportion for categorical variables.

Abbreviations: ACEI/ARB, angiotensin‐converting enzyme inhibitor/angiotensin receptor blocker; BMD, bone mineral density; BMI, body mass index; CCB, calcium channel blockers; CVD, cardiovascular disease; DPN, diabetic peripheral neuropathy; eGFR, estimated glomerular filtration rate; FN, femoral neck; HbA1_C_, glycated hemoglobin; HDL, high‐density lipoprotein; L2‐4, lumbar spine; LDL, low‐density lipoprotein; TH, total hip.

### 3.2 Restricted cubic spline (RCS) curves analysis

**FIGURE 2:**
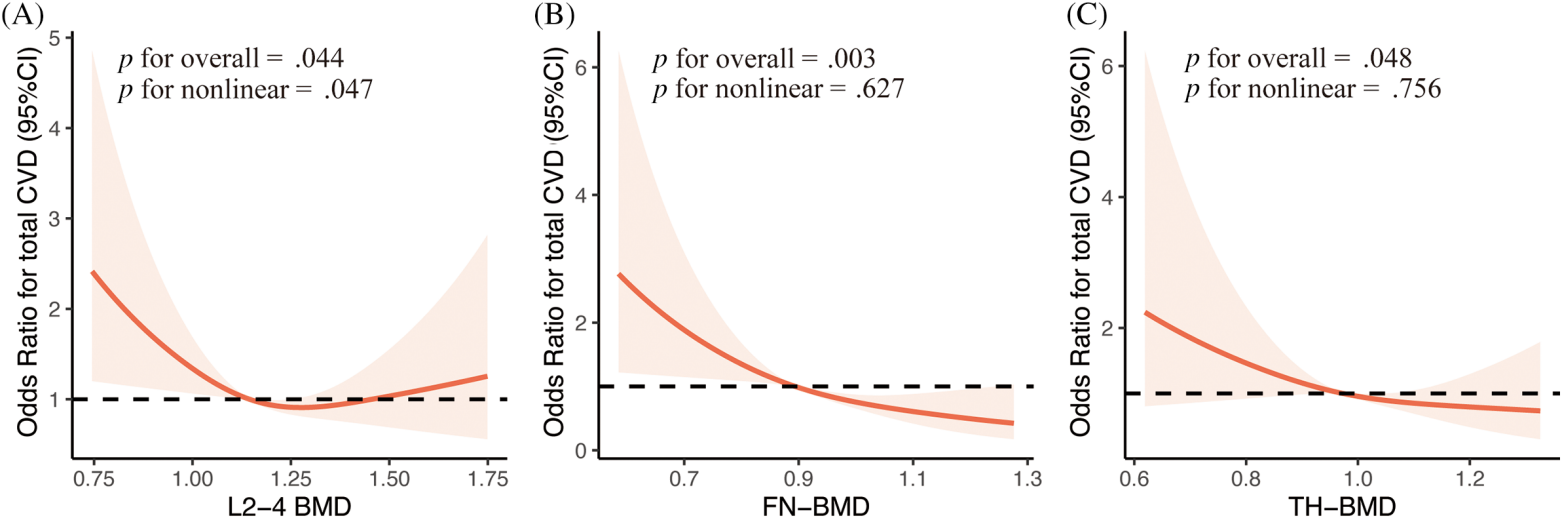
Restricted cubic spline of the association between BMD and CVD events in men with T2DM. The gray line represents the OR = 1. The red line shows the OR value. (A) The RCS curve of L2‐4 BMD (g/cm^2^) and the prevalence of CVD in men. (B) The RCS curve of FN‐BMD (g/cm^2^) and the prevalence of CVD in men. (C) The RCS curve of TH‐BMD (g/cm^2^) and the prevalence of CVD in men. BMD, bone mineral density; CVD, cardiovascular disease; FN, femoral neck; OR, odds ratio; RCS, restricted cubic spline; TH, total hip.

Figure 2 depicts the restricted cubic spline curves showing the relationship between the BMD (g/cm^2^) and CVD events. Among them, the BMDs at the FN (*p* < .01) and TH (*p* < .05) were negatively correlated with CVD events. Furthermore, there was a nonlinear relationship between L2‐4‐BMD and CVD events (*p* for nonlinearity = .047).

### 3.3 Association between BMDs and CVD events

The findings of logistic regression analysis for the relationship between the BMDs and CVD events are shown in Table 2. The unadjusted ORs of CVD events for 1‐SD decrease in BMDs at the L2‐4, FN, and TH were 1.22 (95% confidence interval [CI] [1.02–1.45]), 1.22 (95% CI [1.00–1.48]), and 1.13 (95% CI [0.94–1.36]), respectively (model 1). After complete adjustments for confounding covariates, for each 1‐SD decrease in BMD of L2‐4, FN, and TH, the ORs were 1.34 (95% CI [1.11–1.61], *p* < .05), 1.30 (95% CI [1.05–1.60], *p* < .05), and 1.26 (95% CI [1.02–1.55], *p* < .05), respectively (model 3). In particular, T‐scores of < −1 for the L2‐4 (*p* < .05) and FN (*p* < .05), but not for the TH (*p* = .384), were independently associated with CVD events, even after adjustment for age and other clinical risk factors (Table 3). The clinical diagnosis of osteopenia/osteoporosis based on the combined T‐score was also independently associated with CVD events.

**TABLE 2:** Associations between BMDs (g/cm^2^) and the prevalence of CVD events.

	Model 1			Model 2		Model 3	
	SD	OR (95% CI)	*p* value	OR (95% CI)	*p* value	OR (95% CI)	*p* value
L2‐4 BMD (per 1‐SD decrease)	0.189	1.22 (1.02–1.45)	.025	1.3 (1.08–1.55)	.005	1.34 (1.11–1.61)	.002
FN BMD (per 1‐SD decrease)	0.136	1.22 (1.00–1.48)	.047	1.3 (1.06–1.6)	.012	1.3 (1.05–1.60)	.017
TH BMD (per 1‐SD decrease)	0.138	1.13 (0.94–1.36)	.209	1.24 (1.01–1.52)	.036	1.26 (1.02–1.55)	.032

*Note*: Model 1: age; Model 2: model 1 variables plus smoker, alcohol user, BMI, diabetes duration, and hypertension; Model 3 was adjusted for Model 2 variables plus DPN, diabetic retinopathy, HbA1c, eGFR, HDL, LDL, insulin use, ACEI/ARB, β‐blocker, CCB, diuretic, aspirin and hypolipidemic drugs.

Abbreviations: ACEI/ARB, angiotensin‐converting enzyme inhibitor/angiotensin receptor blocker; BMD, bone mineral density; BMI, body mass index; CCB, calcium channel blockers; CI, confidence interval; CVD, cardiovascular disease; DPN, diabetic peripheral neuropathy; eGFR, estimated glomerular filtration rate; FN, femoral neck; HbA1_C_, glycated hemoglobin; HDL, high‐density lipoprotein; L2‐4, lumbar spine; LDL, low‐density lipoprotein; OR, odds ratio; TH, total hip.

**TABLE 3:** Associations between osteopenia/osteoporosis and prevalence of CVD events.

Diagnosis of osteopenia and osteoporosis based on the T‐score			Model 1		Model 2		Model 3	
L2‐4 T‐score	Number	CVD *n* (%)	OR (95% CI)	*p* value	OR (95% CI)	*p* value	OR (95% CI)	*p* value
T > −1	648	115 (17.7)	Ref.		Ref.		Ref.	
T ≤ −1	208	48 (23.1)	1.3 (0.87–1.96)	.199	1.55 (1.02–2.37)	.04	1.65 (1.06–2.55)	.026
FN T‐score								
T > −1	686	115 (16.8)	Ref.		Ref.		Ref.	
T ≤ −1	170	48 (28.2)	1.5 (0.98–2.28)	.059	1.66 (1.07–2.58)	.023	1.70 (1.08–2.67)	.022
TH T‐score								
T > −1	786	148 (18.8)	Ref.		Ref.		Ref.	
T ≤ −1	70	15 (21.4)	1.01 (0.53–1.92)	.969	1.2 (0.61–2.34)	.598	1.36 (0.68–2.72)	.384
Combined T‐score				.115		.024		.025
T > −1	576	93 (16.1)	Ref.		Ref.		Ref.	
−2.5 < T ≤ −1	255	62 (24.3)	1.49 (1.02–2.2)	.042	1.67 (1.12–2.5)	.011	1.69 (1.12–2.56)	.012
T ≤ −2.5	25	8 (32)	1.41 (0.56–3.52)	.465	2.06 (0.8–5.3)	.133	2.12 (0.79–5.7)	.135

*Note*: Model 1: age; Model 2: model 1 variables plus smoker, alcohol user, BMI, diabetes duration, and hypertension; Model 3: was adjusted for model 2 variables plus DPN, diabetic retinopathy, HbA1c, eGFR, HDL, LDL, insulin use, ACEI/ARB, β‐blocker, CCB, diuretic, aspirin and hypolipidemic drugs.

Abbreviations: ACEI/ARB, angiotensin‐converting enzyme inhibitor/angiotensin receptor blocker; BMD, bone mineral density; BMI, body mass index; CCB, calcium channel blockers; CI, confidence interval; CVD, cardiovascular disease; DPN, diabetic peripheral neuropathy; eGFR, estimated glomerular filtration rate; FN, femoral neck; HbA1_C_, glycated hemoglobin; HDL, high‐density lipoprotein; L2‐4, lumbar spine; LDL, low‐density lipoprotein; OR, odds ratio; TH, total hip.

### 3.4 Association between previous fracture and CVD events

Among the total participants, 32 (3.7%) individuals were in the previous fracture group, 14 of them had CVD (Table 4). Individuals with previous fracture had a higher likelihood of developing CVD events than those without. Even after adjustment for underlying confounding variables, the previous fracture was independently associated with CVD events and OR with a 95% CI of 2.93 (1.25–6.86).

**TABLE 4:** Adjusted ORs for associations between osteoporotic fracture and the prevalence of CVD events in men with T2DM.

Osteoporotic fracture	Total CVD	Non‐CVD	Model 1	Model 2	Model 3
			OR (95% CI)	OR (95% CI)	OR (95% CI)
No	149	675	Ref.	Ref.	Ref.
Yes	14	18	2.74 (1.25–6.02)	2.84 (1.26–6.4)	2.93 (1.25–6.86)
*p* value			.012	.012	.013

*Note*: Model 1: age; Model 2: model 1 variables plus smoker, alcohol user, BMI, diabetes duration, and hypertension; Model 3 was adjusted for Model 2 variables plus DPN, diabetic retinopathy, HbA1c, eGFR, HDL, LDL, insulin use, ACEI/ARB, β‐blocker, CCB, diuretic, aspirin and hypolipidemic drugs.

Abbreviations: ACEI/ARB, angiotensin‐converting enzyme inhibitor/angiotensin receptor blocker; BMD, bone mineral density; BMI, body mass index; CCB, calcium channel blockers; CI, confidence interval; CVD, cardiovascular disease; DPN, diabetic peripheral neuropathy; eGFR, estimated glomerular filtration rate; HbA1_C_, glycated hemoglobin; HDL, high‐density lipoprotein; LDL, low‐density lipoprotein; OR, odds ratio; T2DM, type 2 diabetes mellitus.

### 3.5 Subgroup analyses

Subgroup analyses stratified by age, BMI, diabetes duration, hypertension, DPN, and diabetic retinopathy are shown in Table 5. Results showed that 1‐SD decrease in BMDs of the L2‐4, FN, and TH in almost all subgroups was associated with a higher risk for CVD events. Specifically, the decrease of BMD at the L2‐4 was an independent risk factor for CVD events in almost all subgroups. In contrast, FN‐BMD decrease was an independent risk factor for CVD events in participants older than 60 years, diabetes duration over 10 years, BMI less than 25 kg/m^2^, hypertension, and no diabetic retinopathy (*p* < .05). Furthermore, there was a significant interaction for associations between FN‐BMD and CVD in the subgroup analyses of hypertension. For per 1‐SD reduction in FN‐BMD, the risk of CVD events in the hypertension subgroup increased by 55%.

**TABLE 5:** Subgroup analyses for the risk of CVD events per 1‐SD decrease in BMD (g/cm^2^) in men with T2DM.

Subgroups	*N*	Total CVD (%)	L2‐4 BMD (per 1‐SD decrease)	FN BMD (per 1‐SD decrease)	TH BMD (per 1‐SD decrease)
			Fully adjusted OR (95% CI)	Fully adjusted OR (95% CI)	Fully adjusted OR (95% CI)
Age			Interaction *p* = .488	Interaction *p* = .507	Interaction *p* = .669
<60 years	589	73 (12.4)	1.41 (1.03–1.93)*	1.24 (0.93–1.66)	1.3 (0.96–1.75)
≥60 years	267	90 (33.7)	1.4 (1.08–1.81)*	1.50 (1.06–2.12)*	1.34 (0.97–1.85)
BMI			Interaction *p* = .616	Interaction *p* = .689	Interaction *p* = .603
<25 kg/m^2^	437	77 (17.6)	1.36 (1.01–1.83)*	1.52 (1.08–2.13)*	1.40 (1.02–1.93)*
≥25 kg/m^2^	419	86 (20.5)	1.43 (1.09–1.87)*	1.1 (0.82–1.49)	1.09 (0.80–1.48)
Diabetes duration			Interaction *p* = .661	Interaction *p* = .537	Interaction *p* = .919
<10 years	473	65 (13.7)	1.39 (0.98–1.97)	1.27 (0.9–1.79)	1.38 (0.97–1.97)
≥10 years	383	98 (25.6)	1.42 (1.11–1.82)**	1.38 (1.03–1.85)*	1.31 (0.98–1.75)
Hypertension			Interaction *p* = .21	Interaction *p* = .029	Interaction *p* = .092
No	420	50 (11.9)	1.44 (1.01–2.04)*	1.14 (0.78–1.67)	1.17 (0.79–1.72)
Yes	436	113 (25.9)	1.44 (1.13–1.82)**	1.55 (1.17–2.05)**	1.40 (1.07–1.82)*
DPN			Interaction *p* = .697	Interaction *p* = .911	Interaction *p* = .763
No	546	81 (14.8)	1.4 (1.05–1.87)*	1.25 (0.93–1.69)	1.27 (0.97–1.72)
Yes	310	82 (26.5)	1.25 (0.97–1.62)*	1.29 (0.94–1.76)	1.19 (0.88–1.62)
Diabetic retinopathy			Interaction *p* = .441	Interaction *p* = .858	Interaction *p* = .854
No	684	111 (16.2)	1.28 (1.02–1.61)*	1.30 (1.01–1.68)*	1.27 (0.996–1.63)
Yes	172	52 (30.2)	1.52 (1.06–2.19)*	1.40 (0.84–2.02)	1.18 (0.76–1.83)

*Note*: Fully adjusted OR: age, smoker, alcohol user, BMI, diabetes duration, hypertension, DPN, diabetic retinopathy, HbA1c, eGFR, HDL, LDL, insulin use, ACEI/ARB, β‐blocker, CCB, diuretic, aspirin and hypolipidemic drugs.

Abbreviations: ACEI/ARB, angiotensin‐converting enzyme inhibitor/angiotensin receptor blocker; BMD, bone mineral density; BMI, body mass index; CCB, calcium channel blockers; CI, confidence interval; CVD, cardiovascular disease; DPN, diabetic peripheral neuropathy; eGFR, estimated glomerular filtration rate; FN, femoral neck; HbA1_C_, glycated hemoglobin; HDL, high‐density lipoprotein; L2‐4, lumbar spine; LDL, low‐density lipoprotein; OR, odds ratio; TH, total hip; T2DM, type 2 diabetes mellitus.

* *p* < .05;

** *p* < .01.

## 4 DISCUSSION

This study explored the relationship between BMD and CVD events in men with T2DM. Our findings demonstrated a significant inverse association between BMD levels, particularly at FN, and the prevalence of CVD events. These associations remained statistically significant even after adjusting for established CVD risk factors. Specifically, 1‐SD decrease in BMD at L2‐4, FN, and TH in men with T2DM was associated with a 1.34‐fold, 1.3‐fold, and 1.26‐fold increased risk of total CVD events after full adjustment. Additionally, we observed that previous fracture was independently associated with an increased risk of CVD.

Although previous studies have hinted at a potential link between BMD and CVD, most of these have predominantly focused on women in the general population.^21^, ^22^ In contrast, our study was unique in delving into the connection between BMD and the risk of CVD events in men, particularly those with T2DM. In nondiabetic patients, our prior research revealed an association between lower forearm ultrasonography BMD and a 10‐year risk of CVD, emphasizing the predictive potential of BMD for CVD.^23^ Compared with prior studies in older women, studies have shown that the magnitude of both any clinical fracture and hip fracture associated with diabetes among older men was comparable to that reported in older women and that a large portion of this risk was explained by diabetes‐related comorbidities.^24^ Other research results showed that the development of diabetic retinopathy may involve the progress of bone formation and resorption defects induced by T2DM.^25^ It is important to note that CVD stands as the foremost cause of mortality in individuals with T2DM.^26^ In light of this, we embarked on a comprehensive exploration of the relationship between BMD and CVD events across multiple anatomical sites and the interplay between fracture and CVD. Our investigation unveiled the independent and incremental value of low BMD, particularly at the FN, and previous fracture in assessing the risk of CVD in men with T2DM.

Our findings in men were an essential addition to the existing literature. Similar to previous studies, our study demonstrated that lower BMD was significantly associated with CVD events, independent of the traditional CVD risk factors, such as age, obesity, hypertension, glycemia control, dyslipidemia, smoking, and alcohol habits. BMD, typically measured by DXA, was a well‐established tool for diagnosing osteoporosis, assessing fracture risk, and monitoring osteoporosis therapy.^27^ Recent studies have suggested that BMD holds promise in assessing CVD risk. For instance, a prospective cohort study in Korea found that low BMD was associated with an increased risk of major adverse cardiovascular events in predialysis chronic kidney disease patients.^28^ Similarly, an analysis in a biracial cohort demonstrated a significant association between volumetric BMD at the spine and CVD risk in white men.^29^ However, few studies have investigated the possible relationship between BMD and the expected risk of CVD events in men, especially in patients with diabetes. Therefore, our study filled this gap, and the results showed that the clinical diagnosis of osteopenia or osteoporosis in men with T2DM provided independent prognostic value for CVD events.

Intriguingly, the restricted cubic spline results of the FN‐BMD demonstrated the most substantial connection with CVD events in men with T2DM. There was evidence that men with long‐term T2DM developed significant iliac artery calcification and decreased BMD of the FN.^30^ Therefore, BMD reduction at FN might be an effective marker for CVD risk than other sites in men with diabetes. In contrast, the relationship between lumbar‐spine BMD and CVD events seemed to be nonlinear, possibly due to factors such as lumbar osteophyte, facet osteoarthritis, vertebral fracture, and ligament calcification,^31^ which might interfere with accurate DXA measurement of vertebral BMD, showing pseudo‐increased BMD.^32^

Although the precise mechanisms linking low BMD to CVD have remained under investigation, some common pathways were proposed. Abnormal bone remodeling and increased bone resorption may lead to excessive calcium release from bone mass, resulting in hypercalcemia.^33^ Elevated calcium levels were associated with an increased risk of CVD.^34^ Specifically, subjects with low BMD also exhibited a higher prevalence of vascular calcifications and subclinical atherosclerosis.^35^, ^36^

An underlying mechanism of low BMD might explain the relationship between previous fracture and CVD events. Although some studies demonstrated that the risk of fracture in T2DM patients was higher than in the average population at any given BMD,^37^, ^38^ the age‐adjusted risk ratios for hip fracture and nonspinal fractures per SD decrease in BMD were similar between people with T2DM and those without.^39^ In addition, reduced physical activity was common after fractures in patients with T2DM due to slow recovery,^40^, ^41^ which might be a key risk factor for CVD.^42^, ^43^

Subgroup analyses consistently supported the association between BMD and CVD events across various subgroups, with the 1‐SD decrease in BMDs at the L2‐4, FN, and TH associated with a higher risk for CVD events for almost all subgroups. However, a significant interaction was observed in the hypertension subgroup, suggesting that hypertension might influence the relationship between FN‐BMD and CVD events. Studies have shown that high blood pressure was associated with increased urinary calcium loss, which could disrupt the calcium balance needed for bone remodeling.^44^ Moreover, epidemiological investigations have found that the increase in blood pressure was related to the increased rate of bone mineral loss.^45^ More research would be required to investigate this connection and identify the underlying mechanisms. Likewise, a previous study reported considerably lower bone loss at the lumbar spine than at the FN in older men with T2DM.^46^ Bone loss patterns may differ between these two skeletal sites,^47^ which could lead to discrepancies in skeletal status.^48^ Therefore, these results implied that among diabetic men, a decline in FN‐BMD may signal an elevated risk of CVD. Overall, our results demonstrated the potential that BMD could be a valuable biomarker for determining the likelihood of CVD events in individuals with T2DM. Further studies should investigate the putative underlying mechanisms of this connection and the possibility that BMD‐improving therapies could lower the incidence of CVD events in this population.

Nevertheless, our study had several limitations due to its cross‐sectional nature. First, we did not measure changes in BMD over time and thus failed to determine whether their changes were involved in the associations. The evaluation of causality was also limited. Second, as a retrospective study, recall bias cannot be avoided, although we tried our best to help patients recall the details of CVD events and then search for evidence of disease diagnosis in the EHRs. Third, we did not evaluate any parameters related to inflammation or disturbed calcium metabolism, potential mediators between bone and vascular disease. Finally, the patients in the current cohort were enrolled 10 years ago. Using the latest data, we will explore and compare the relationship between bone metabolism and CVD in T2DM patients.

## 5 CONCLUSION

The prevalence of CVD was inversely correlated with BMD levels in men with T2DM, particularly in the FN. In addition, the previous fracture was independently associated with an increased risk of CVD. We hypothesized that monitoring FN‐BMD and early intervention would help reduce CVD risk in men with T2DM, especially those with hypertension. Future research should elucidate the causal relationship and investigate the underlying mechanisms.

## AUTHOR CONTRIBUTIONS

Bei Tao, Li‐hao Sun, and Jian‐min Liu contributed to the study's conception and design. Material preparation and data collection were performed by Xiao‐ke Kong, Rui Xie, and Xiao‐feng Wang. Xiao‐ke Kong and Rui Xie analyzed data and wrote the first manuscript. Jie‐li Lu participated in manuscript revision. All authors were involved in revising the manuscript and had final approval of the submitted and published versions. Xiao‐ke Kong and Rui Xie contributed equally to this work and shared the first authorship.

## FUNDING INFORMATION

This work was supported by diabetes mellitus research fund program from Shanghai Medical and Health development foundation (DMRFP_II_06 from SHMHDF) and the CMA·Young and Middle‐aged Doctors Outstanding Development Program‐Osteoporosis Specialized Scientific Research Fund Project (GX2021B01).

## CONFLICT OF INTEREST STATEMENT

Jian‐min Liu is an Editorial Board member of the *Journal of Diabetes* and a coauthor of this article. To minimize bias, he was excluded from all editorial decision‐making related to the acceptance of this article for publication.

## Data Availability

The datasets used and/or analyzed during the present study are available from the corresponding author on reasonable request.
